# Rational design of new NO and redox sensitivity into connexin26 hemichannels

**DOI:** 10.1098/rsob.140208

**Published:** 2015-02-11

**Authors:** Louise Meigh, Daniel Cook, Jie Zhang, Nicholas Dale

**Affiliations:** School of Life Sciences, University of Warwick, Coventry CV4 7AL, UK

**Keywords:** nitrosylation, gap junction, hemichannel, intracellular redox

## Abstract

CO_2_ directly opens hemichannels of connexin26 (Cx26) by carbamylating K125, thereby allowing salt bridge formation with R104 of the neighbouring subunit in the connexin hexamer. The formation of the inter-subunit carbamate bridges within the hexameric hemichannel traps it in the open state. Here, we use insights derived from this model to test whether the range of agonists capable of opening Cx26 can be extended by promoting the formation of analogous inter-subunit bridges via different mechanisms. The mutation K125C gives potential for nitrosylation on Cys125 and formation of an SNO bridge to R104 of the neighbouring subunit. Unlike wild-type Cx26 hemichannels, which are insensitive to NO and NO_2_^−^, hemichannels comprising Cx26^K125C^ can be opened by NO_2_^−^ and NO donors. However, NO_2_^−^ was unable to modulate the doubly mutated (K125C, R104A) hemichannels, indicating that an inter-subunit bridge between C125 and R104 is required for the opening action of NO_2_^−^. In a further test, we introduced two mutations into Cx26, K125C and R104C, to allow disulfide bridge formation across the inter-subunit boundary. These doubly mutated hemichannels open in response to changes in intracellular redox potential.

## 2. Introduction

The human genome contains 21 connexin genes [1]. Connexins are one of the main gene families that can form gap junctions. Each gap junction is made of two hemichannels, comprising six connexin subunits, one in each plasma membrane of two adjacent cells. The hemichannels dock to form a continuous passageway linking the cytoplasm of the coupled cells that permits transfer of ions and small molecules. In addition to this canonical function of connexins as gap junctions, undocked hemichannels can also have physiological functions [2,3]. We have shown that hemichannels of three closely related *β* connexins, Cx26, 30 and 32, can be opened by CO_2_ [4]. In the case of Cx26, we have established that this is a direct action in which CO_2_ binds covalently to K125 via a carbamylation reaction [5]. The carbamylated residue can then form a salt bridge to the neighbouring subunit in the hexamer [5]. This opens the hemichannel, and allows release of ATP, which is an important biological signalling molecule [6]. The CO_2_-dependent opening of Cx26, and consequent release of ATP, is a key mechanism in the regulation of breathing by CO_2_ [6].

**Figure 1.**
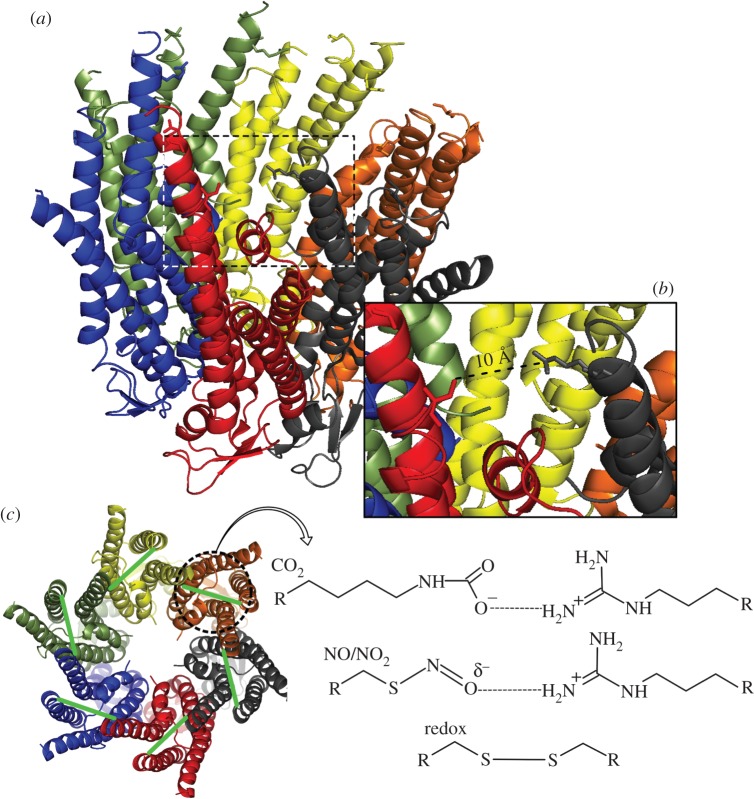
The structure of Cx26^K125C^ based on structure 2zw3 showing the potential interaction of C125 and R104. (*a*) The entire hemichannel showing C125 and R104 in each subunit. (*b*) Inset showing the distance between C125 and R104 at one inter-subunit boundary. (*c*) View of Cx26 from the cytoplasmic side. The green lines show the location of the inter-subunit bridges between residues 125 and 104, which occur during carbamylation of K125 by CO_2_ (top scheme on the right [5]). This paper tests whether nitrosylation of C125 can form a bridge to R104 (middle scheme on the right), and whether a disulfide bridge can form between C125 and C104 of adjacent subunits (bottom scheme on the right).

In this paper, we demonstrate that it is possible to increase the range of molecules that can alter the gating of Cx26 via a rational mutational strategy. Given that formation of a bridge between residues 125 and 104 in adjacent subunits seems vital for CO_2_-mediated hemichannel opening [5], we have explored whether alternative mechanisms of bridge formation might also be effective. NO is an important gaseous signalling molecule that is produced by cells and can act via activation of a guanylate cyclase. NO can also signal via a nitrosylation reaction, whereby cysteine residues become converted to SNO groups (figure 1) [7]. We have tested whether the mutation K125C has the ability to support NO/NO_2_^−^-mediated channel opening. If C125 were to be nitrosylated, the resulting SNO group would have the potential to form an inter-subunit salt bridge with Arg104 [5] (figure 1) and, if our model is correct, Cx26^K125C^ should be opened by NO/NO_2_^−^. We have additionally demonstrated, in hemichannels with the mutations K125C and R104C, that disulfide bridge formation between subunits will allow hemichannel opening in response to changes in intracellular redox.

## 3. Methods

### 3.1. Mutant connexin production

Cx26^K125C^ and Cx26^K125C,R104C^ mutants were produced from wild-type Cx26 in a Puc19 plasmid via the Quikchange protocol. The primers used were as follows: Cx26^K125C^ forward, 5′ CGA AGA GAT CAA AAC CCA GTG CGT CCG TAT CGA AGG GTC CC 3′; reverse, 5′ GGG ACC CTT CGA TAC GGA CGC ACT GGG TTT TGA TCT CTT CG 3′; for Cx26^K125C,R104A^, a second set of primers was used to introduce the R104A mutation into Cx26^K125C^—forward, 5′ GGC CTA CCG GAG ACA CGA AAA GAA AGC GAA GTT CAT GAA GG 3′; reverse, 5′ CCT TCA TGA ACT TCG CTT TCT TTT CGT GTC TCC GGT AGG CC 3′; and for Cx26^K125C,R104C^ a second primer set to introduce the R104C mutation into Cx26^K125C^—forward, 5′ CCG GAG ACA CGA AAA GAA ATG CAA GTT CAT GAA GGG AGA G 3′; reverse, 5′ CTC TCC CTT CAT GAA CTT GCA TTT CTT TTC GTG TCT CCG G 3′. The DNA produced was processed using DPN1 digest and bacterial transformation. DNA mutation was confirmed by genomic sequencing. The Cx26 mutants were subcloned into a pCAG-GS vector for mammalian cell transfection.

### 3.2. HeLa cell culture

HeLa cells were cultured by standard methods in DMEM and 10% FCS with addition of 3 mM CaCl_2_. For experimentation, cells were plated onto coverslips at a density of 5 × 10^4^ cells per well. Transient transfections were performed using the Genejuice protocol.

### 3.3. Dye loading protocols

#### 3.3.1. Solutions used

Control saline: 124 mM NaCl, 26 mM NaHCO_3_, 1.25 mM NaH_2_PO_4_, 3 mM KCl, 10 mM d-glucose, 1 mM MgSO_4_, 1 mM CaCl_2_.

Zero Ca^2+^ saline: 124 mM NaCl, 26 mM NaHCO_3_, 1.25 mM NaH_2_PO_4_, 3 mM KCl, 10 mM d-glucose, 1 mM MgSO_4_, 1 mM MgCl_2_, 1 mM EGTA.

All solutions were saturated with 95% O_2_/5% CO_2_.

**Figure 2.**
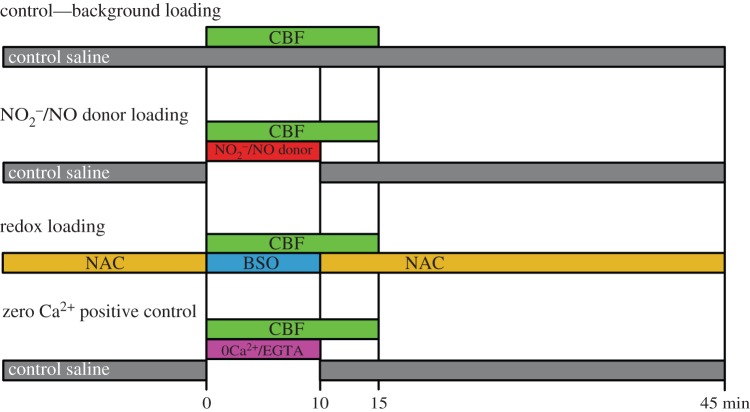
Dye loading protocols used to test the sensitivity of HeLa cells expressing wild-type and mutant Cx26 hemichannels to nitrite, NO donors and changes in redox potential. NAC, *N*-acetyl l-cysteine; BSO, l-buthionine sulfoximine; CBF, carboxyfluorescein.

Figure 2 summarizes in diagrammatic form the dye loading protocols used to assess NO_2_^−^/NO and redox sensitivity. The protocols are based on those that we have previously validated for determining the CO_2_ sensitivity of Cx26^WT^ hemichannels [4,5,8].

#### 3.3.2. Sensitivity to NO_2_^−^ and NO donors

Coverslips plated with HeLa cells transiently transfected with Cx26^K125C^, Cx26^K125C,R104A^ or Cx26^WT^ were exposed to control saline containing 200 µM carboxyfluorescein (CBF) and 100 µM NaNO_2_ or the NO donors 2-(*N*,*N*-diethylamino)-diazenolate 2-oxide (DEA-NONOate, 100 or 10 µM) or sodium nitroprusside (SNP, 100 µM), for 10 min. This was followed by saline with 200 µM CBF for 5 min to allow the hemichannels to close in the absence of a stimulus and thus reduce loss of dye from the cells through any open hemichannels, and a 30 min wash with saline (in the absence of CBF) to ensure that all dye was removed from the outside of the cells. The cells were then immediately imaged to quantify dye loading.

To establish whether the guanylate cyclase blocker H-[1,2,4]oxadiazolo[4,3-a]quinoxalin-1-one (ODQ) could affect the modulation of Cx26^K125C^ by DEA-NONOate, ODQ at 3 µM was pre-applied in control saline for 15 min, and present during the application of the DEA-NONOate in the presence of CBF. This was then followed by the washing procedure as described above.

The control comparison used to establish any baseline loading in the absence of a stimulus followed exactly the same protocol, but no NaNO_2_ or NO donors were present. Thus, the HeLa cells were exposed to 200 µM CBF in control saline for 15 min, followed by 30 min of washing.

The zero Ca^2+^-positive control involved exposure of Cx26 (mutant or wild-type) expressing cells to saline containing 200 µM CBF in zero Ca^2+^ saline for 10 min. This was followed by control saline with 200 µM CBF for 5 min and 30 min of washing with saline. We have previously shown that non-transfected HeLa cells, or HeLa cells transfected with an empty vector have no responsiveness to the zero Ca^2+^ stimulus [5,8]. Only batches of transfected cells that successfully demonstrated dye loading in response to zero Ca^2+^ were included in the analysis.

#### 3.3.3. Redox sensitivity

We used two solutions to manipulate intracellular redox: ‘reduced saline’ contained 4 mM *N*-acetyl-l-cysteine (NAC) and ‘oxidized saline’ contained 1 mM l-buthionine sulfoximine (BSO). The HeLa cells were exposed for 5 min to reduced saline, and then transferred to oxidized saline plus 200 µM CBF for 10 min. They were then washed with reduced saline plus CBF for 5 min before being washed for 30 min in reduced saline.

The no-stimulus control to assess baseline loading was exposure of the cells to 200 µM CBF in reduced saline for 15 min, followed by 30 min of washing in reduced saline.

Each experiment was independently repeated (i.e. separate transfections on different batches of cells on different days) at lease five times as noted in the relevant figure legends.

### 3.4. Imaging and analysis

The cells were imaged in their living state by epifluorescence (Scientifica Slice Scope, Cairn Research OptoLED 470 nm illumination, 60× water Olympus immersion objective, NA 1.0, Hamamatsu ImageEM EMCCD camera, Metafluor software). Using ImageJ, the extent of dye loading was measured by drawing a region of interest (ROI) around individual cells and calculating the mean pixel intensity for the ROI. The mean pixel intensity of the background fluorescence was also measured in a representative ROI, and this value was subtracted from the measures obtained from the cells. All of the images displayed in the figures reflect this procedure in that the mean intensity of the pixels in a representative background ROI has been subtracted from every pixel of the image. At least 40 cells were measured in each condition per independent repetition. The mean pixel intensities from all independent repetitions were plotted in one cumulative probability distribution. Statistical comparisons of dye loading under different conditions were performed by comparing the individual median values from each independent repetition via the Mann–Whitney *U*-test. As we have used non-parametric statistical comparisons, we show the median values together with the upper and lower quartiles in the figures.

### 3.5. Patch clamp recordings

Coverslips containing non-confluent cells were placed into a perfusion chamber at 28°C in sterile filtered standard saline. Standard patch clamp techniques were used to make whole cell recordings and outside out or inside out membrane patch recordings. For whole cell patch clamp, the intracellular fluid in the patch pipette contained: K-gluconate 130 mM, KCl 10 mM, EGTA 10 mM, CaCl_2_ 2 mM, HEPES 10 mM, pH 7.3, and adjusted to 295 mOsm with pure H_2_O (dilution no more than 2%). All whole-cell recordings were performed at a holding potential of −40 mV with regular steps of 5 s, every 10 s, to −50 mV to assess whole cell conductance.

## 4. Results

### 4.1. The mutation K125C creates an NO_2_^−^ and NO-sensitive hemichannel

**Figure 3.**
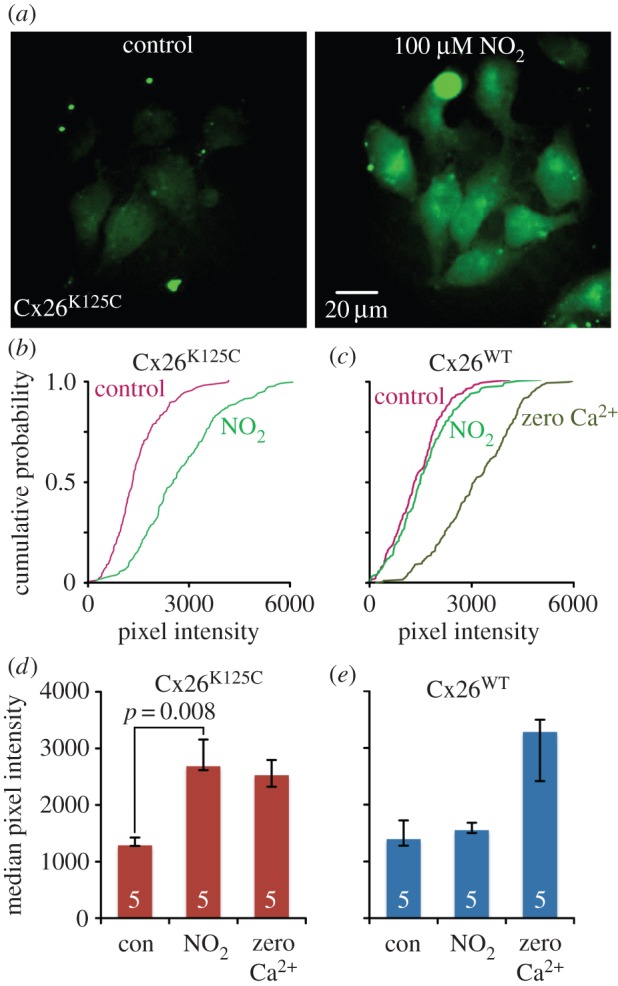
Cx26^K125C^ hemichannels can be opened by NO_2_^−^. (*a*) Images showing HeLa cells expressing Cx26^K125C^. In the control (left), the cells have been exposed to CBF in saline, and very little dye loading is seen. When the cells were exposed to CBF in the presence of 100 µM NO_2_^−^, dye loading was observed. (*b*) Cumulative probability plots of pixel intensity (combined data from five independent repetitions) demonstrate the exposure to NO_2_^−^ cause a rightward shift to higher pixel intensities in HeLa cells expressing Cx26^K125C^. (*c*) HeLa cells expressing wild-type Cx26 (Cx26^WT^) do not exhibit dye loading with NO_2_^−^, nevertheless, the positive control of zero Ca^2+^, which opens hemichannels, results in dye loading of the HeLa cells expressing Cx26^WT^, demonstrating the presence of functional hemichannels in the membrane. Combined data from five independent repetitions. (*d,e*) Histograms of median pixel intensity (with lower and upper quartile error bars) for the effects of 100 µM NO_2_^−^ and zero Ca^2+^ stimuli in Cx26^K125C^ and Cx26^WT^, respectively.

We examined the gating of Cx26 hemichannels via an established assay, whereby loading of CBF into Cx26-expressing HeLa cells is a measure of hemichannel opening [5]. We found that 100 µM nitrite caused dye loading in Cx26^K125C^-expressing HeLa cells (*p* = 0.008 compared with background control, figure 3*a,b,d*). The extent of dye loading in 100 µM nitrite was very similar to that caused by the zero Ca^2+^ positive control, suggesting that the nitrite stimulus was very effective at opening the hemichannels (figure 3*d*).

Importantly, we found that HeLa cells expressing wild-type Cx26 had no endogenous sensitivity to nitrite, as no increase in dye loading was seen (figure 3*c,e*). As a positive control to test for functional hemichannels, we used a zero Ca^2+^ challenge, which we have previously shown has no effect on dye loading in parental HeLa cells which have no connexin expression [5,8]. The zero Ca^2+^ stimulus was very effective at causing dye loading in HeLa cells expressing wild-type Cx26 (figure 3*c,d,e*), demonstrating that functional wild-type hemichannels are present. The lack of effect of NO_2_^−^ must therefore be due to its inability to open the wild-type Cx26 hemichannels. Taken together, these results demonstrate that wild-type Cx26 does not respond to NO_2_^−^ with channel opening, but that this new functionality can be conferred on Cx26 by the mutation K125C.

**Figure 4.**
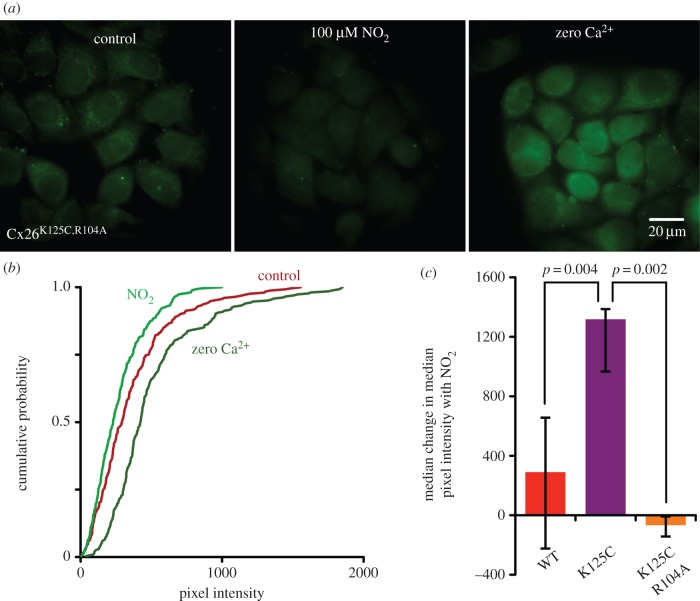
R104 is required for NO_2_^−^ to open Cx26^K125C^ hemichannels. (*a*) Example images showing HeLa cells expressing Cx26^K125C,R104A^. In the control (left), the cells have been exposed to CBF in saline, and very little dye loading is seen. Exposure to 100 µM NO_2_^−^ does not cause dye loading. However, the positive control of removing extracellular Ca^2+^does cause dye loading. Scale bar, 20 µm. (*b*) Cumulative probability plots (combined data from five independent repetitions) show that NO_2_^−^ does not cause dye loading, but the zero Ca^2+^ treatment gives a rightward shift to higher pixel intensities (*p* = 0.048 compared with control, *n* = 5). (*c*) Comparison of the median change in median pixel intensity caused by NO_2_^−^ for HeLa cells expressing wild-type, K125C and K125C, R104A hemichannels. Comparisons via the Mann–Whitney *U*-test (*n* = 5 for K125C and K12C, R104A; *n* = 6 for WT).

We next tested whether R104 was required for the modulatory action of NO_2_^−^, by creating the doubly mutated variant Cx26^K125C,R104A^. We have previously demonstrated that the Cx26^R104A^ is CO_2_ insensitive as the loss of the arginine residue at this location prevents formation of the inter-subunit salt bridge with the carbamylated K125. In this doubly mutated variant, if C125 were to be nitrosylated it would be unable to form a salt bridge to A104. We found that HeLa cells expressing Cx26^K125C,R104A^ did not exhibit dye loading in response to NO_2_^−^ (figure 4*a,b*). However, the positive control of the zero Ca^2+^ stimulus evoked dye loading indicating that functional hemichannels were present (figure 4*a,b*). Overall, while Cx26^K125C^ hemichannels exhibited robust sensitivity to NO_2_^−^, neither Cx26^WT^ nor Cx26^K125C,R104A^ hemichannels displayed any sensitivity to NO_2_^−^ (figure 4*c*). Thus, we conclude that both C125 and R104 are required for nitrite sensitivity.

**Figure 5.**
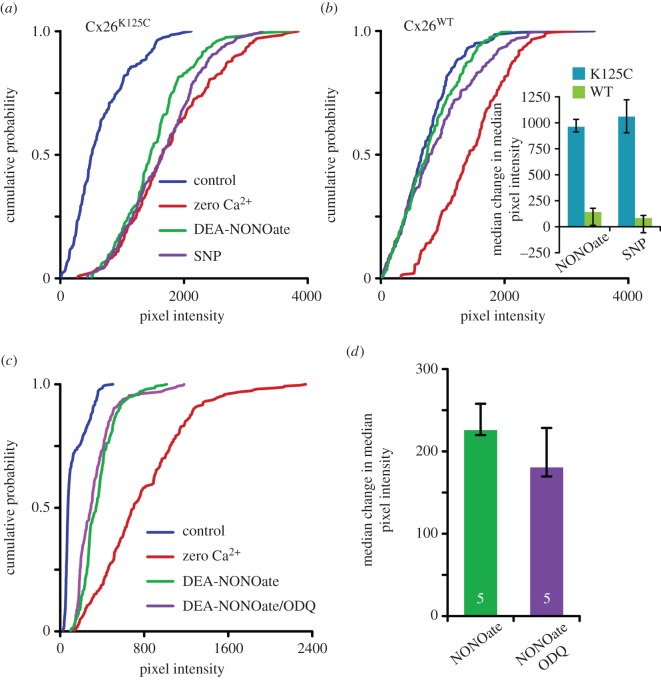
Cx26^K125C^ hemichannels can be opened by NO donors. (*a,b*) Cumulative probability plots of pixel intensity showing the effect of the NO donors SNP and DEA-NONOate (both at 100 µM) on dye loading in HeLa cells expressing Cx26^k125C^ or wild-type Cx26 (Cx26^WT^) compared with the control, and the effect of zero Ca^2+^. The donors do not cause dye loading in HeLa cells expressing Cx26^WT^, but do in HeLa cells expressing Cx26^K125C^—as shown by rightward shift of cumulative probability plots. The cumulative probability plots are from combined data for five independent repetitions of each condition. Inset shows the median change in median pixel intensity (*n* = 5) for the actions of DEA-NONOate and SNP (100 µM) on Cx26^K125C^ and Cx26^WT^. (*c*) A lower dose of DEA-NONOate (10 µM) causes a smaller amount of dye loading compared with the zero Ca^2+^ control. The dye loading is unaffected by the guanylate cyclase inhibitor ODQ (3 µM). (*d*) Summary histogram showing the median change in median pixel intensity caused by 10 µM DEA-NONOate either on its own or with 3 µM ODQ (*n* = 5, *p* = 0.421, Mann–Whitney *U*-test).

NO donors are more usually used to demonstrate activation of the NO signalling pathway and nitrosylation. We therefore additionally tested two NO donors, DEA-NONOate and SNP. Both of these donors at 100 µM caused dye loading into HeLa cells expressing Cx26^K125C^ (figure 5*a*). However, HeLa cells expressing wild-type Cx26 showed no such NO-evoked dye loading (figure 5*b*), indicating that this is a specific effect of the NO donors on HeLa cells expressing Cx26^K125C^. We also tested DEA-NONOate at a lower concentration (10 µM) and found that this too caused dye loading into cells expressing Cx26^K125C^ (*p* = 0.016, *n* = 5, figure 5*c*). However, the extent of the dye loading was less compared with both the higher dose of DEA-NONOate and the zero Ca^2+^ control (figure 5*c*).

In addition to nitrosylation, NO can signal via the activation of guanylate cyclase and the cGMP-dependent protein kinase. We therefore tested whether ODQ, a blocker of guanylate cyclase, affected the modulatory action of DEA-NONOate on HeLa cells expressing Cx26^K125C^. We found that 3 µM ODQ did not affect the degree of dye loading caused by 10 µM DEA-NONOate (figure 5*c,d*). We therefore conclude that guanylate cyclase does not contribute to the modulatory action of the NO donors on Cx26^K125C^.

### 4.2. The mutation K125C, R104C creates a redox-sensitive hemichannel

Disulfide bridges represent a further way in which a bridge between subunits at residues 125 and 104 could form. A disulfide bridge would be sensitive to intracellular redox as this would potentially reduce the disulfide bridge to break it apart or oxidize the cysteine SH groups to promote bridge formation. We therefore made a further variant of Cx26, Cx26^K125C,R104C^, to test whether we could create a redox-sensitive hemichannel.

We used l-BSO and NAC to manipulate the redox state of HeLa cells via altered intracellular concentrations of glutathione [9]. BSO inhibits γ-glutamylcysteine synthase, an enzyme essential for glutathione synthesis [10]. BSO will therefore cause a decrease in the concentration of intracellular glutathione and will make the cell relatively oxidized and promote disulfide bridge formation [9]. Conversely, NAC, by acting as a precursor, can increase the intracellular concentration of cysteine, and thus enhance intracellular glutathione concentrations [11]. NAC will therefore make the cell more reduced and tend to break disulfide bridges [9].

**Figure 6.**
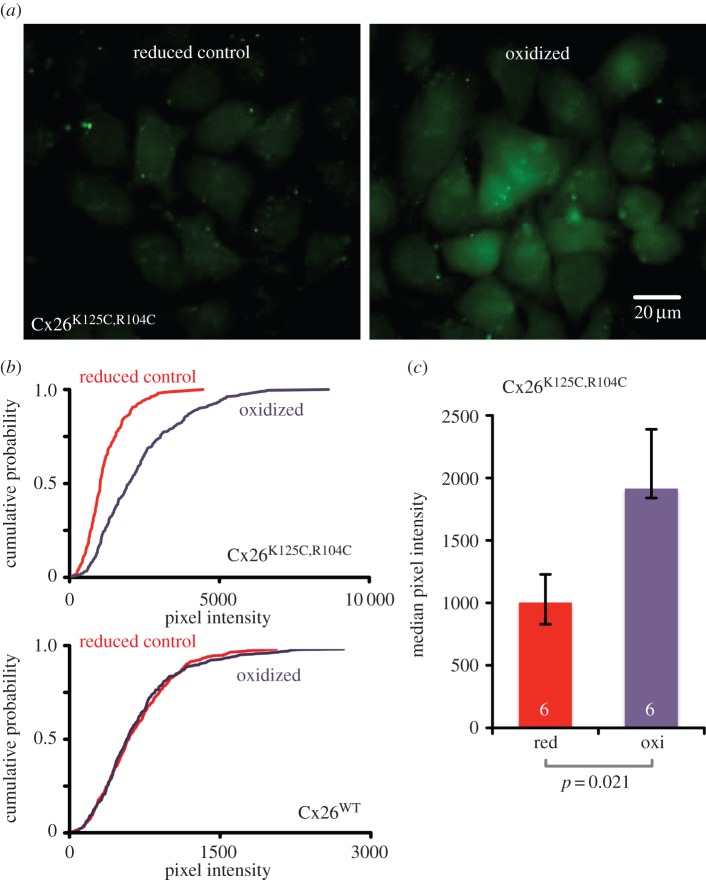
Cx26^K125C,R104C^ hemichannels can be opened by changes in intracellular redox. Intracellular redox was reduced by exposure of cells to saline containing 4 mM NAC, and oxidized by exposure to saline containing 1 mM BSO. (*a*) Images showing CBF loading into HeLa cells expressing Cx26^K125C,R104C^ under reduced control, and oxidized conditions. Under the reduced conditions (hemichannels predicted to be closed), little dye loading was seen. Under oxidized conditions (hemichannels predicted to be open), significant dye loading is seen. (*b*) Cumulative probability plots showing that HeLa cells expressing Cx26^K125C,R104C^ demonstrate a rightward shift in pixel intensity when exposed to oxidized conditions, but cells expressing wild-type Cx26 showed no change in pixel intensity (combined data from six independent repetitions for Cx26^WT^ and Cx26^K125C,R104C^). The Cx26^WT^ cells stably expressed Cx26 and their expression of these hemichannels was verified by examining their sensitivity to CO_2_ with patch clamp recording (figure 7). (*c*) Comparison of median pixel intensity under the reduced and oxidized conditions for HeLa cells expressing Cx26^K125C,R104C^. Comparisons via the Mann–Whitney *U*-test.

We used dye loading in the presence of NAC as a reduced control, and transferred the cells to solutions containing BSO as the oxidized stimulus. Cells exposed to the reduced saline did not exhibit enhanced dye-loading (figure 6). However, when cells were exposed to the oxidized saline, there was a big increase in dye loading compared with control experiments (*p* = 0.021, figure 6). This redox sensitivity is not a property of wild-type Cx26, as HeLa cells stably expressing Cx26 showed no redox-sensitive dye loading (figure 6).

### 4.3. Patch clamp recordings demonstrate the kinetics of agonist sensitivity

**Figure 7.**
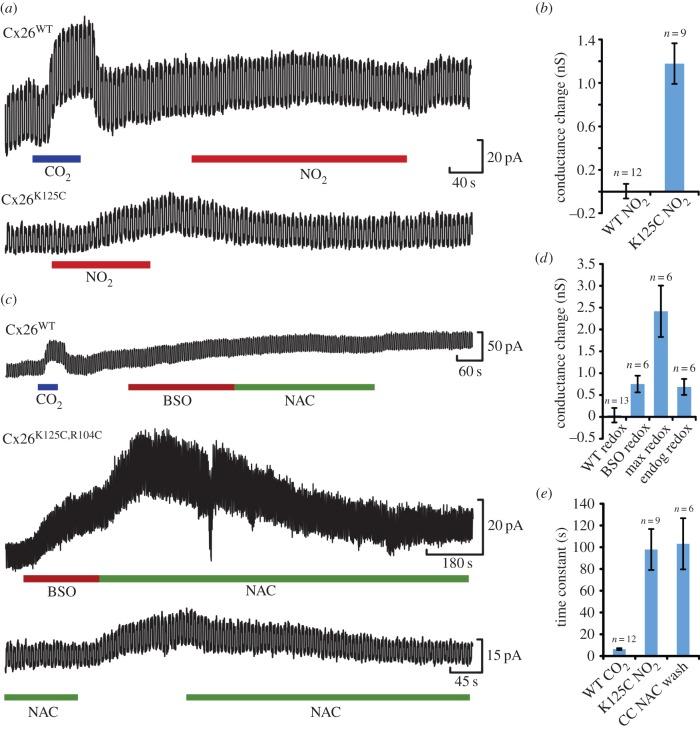
Whole cell patch clamp recordings confirm the sensitivity of Cx26^K125C^ and Cx26^K125C,R104C^ to NO_2_^−^ and intracellular redox respectively. (*a*,*b*) Comparison of effect of 100 µM NO_2_^−^ on HeLa cells expressing Cx26^WT^ and Cx26^K125C^. NO_2_^−^ has no effect on the cells expressing Cx26^WT^, but causes a slow and reversible whole cell conductance increase in cells expressing Cx26^K125C^. Voltage clamp recordings were performed with a holding potential of −40 mV and repeated voltage steps to −50 mV. The size of the current deflection during the voltage step indicates the whole cell conductance. A CO_2_ stimulus given to the Cx26^WT^ cells acted as a positive control to demonstrate functional hemichannels in these cells. (*c*) Comparison of the effect of altering intracellular redox potential of HeLa cells expressing Cx26^WT^ or Cx26^K125C,R104C^. Changes in redox potential had no effect on the cells expressing the wild-type hemichannels (CO_2_ stimulus as a positive control). However, in cells expressing the double mutant Cx26^K125C,R104C^, large reversible increases in whole cell conductance were seen during treatment with BSO and NAC. In cells exposed to NAC, the removal of NAC resulted in a small conductance increase reflecting the difference between the endogenous redox state of the cells and the very reduced state in the presence of NAC. (*d*) Comparison of the mean conductance change of the redox stimulus in the wild-type (WT redox) and Cx26^K125C,R104C^ expressing HeLa cells. For the Cx26^K125C,R104C^ expressing HeLa cells: BSO redox is the whole cell conductance change measured at end of BSO application; max redox is the maximal whole cell conductance change measured during BSO washout with NAC; and endog redox is the whole cell conductance change observed on NAC washout. (*e*) Comparison of the time constant of the increase in whole cell conductance. Current records were fitted with the equation *A*[1 − exp(−*t*/*τ*)] + *B*, where *A* and *B* are constants to appropriately scale the curve to the observed records and τ is the time constant for the change in membrane current. This revealed that the changes evoked by CO­_2_ had a mean time constant of around 5 s, whereas those evoked in Cx26^K125C^ and Cx26^K125C,R104C^, respectively, by NO_2_^−^ (K125C NO_2_) and washout of NAC (CC NAC wash) were some 20 times slower. Error bars represent the s.e.m.

To confirm the dye loading experiments, we performed whole cell patch clamp recordings from HeLa cells expressing wild-type Cx26, Cx26^K125C^ and Cx26^K125C,R104C^. HeLa cells expressing wild-type Cx26 reliably responded to a CO_2_ challenge (figure 7). This response was rapid, having a time course that could be fitted with an exponential function with a time constant of around 5 s. HeLa cells expressing wild-type Cx26 showed no conductance changes in response to NO_2_^−^, BSO or NAC (figure 7). Thus, as we saw with dye loading, the wild-type Cx26 hemichannel has no sensitivity to these compounds.

HeLa cells expressing Cx26^K125C^ responded to 100 µM NO_2_^−^ (figure 7*a,b*). Interestingly, this response was some 20 times slower in onset than the response of wild-type Cx26 to CO_2_ (figure 7*e*). HeLa cells expressing Cx26^K125C,R104C^ exhibited a slow conductance increase when exposed to BSO (figure 7*c,d*). When transferred to NAC, the conductance increase initially accelerated before diminishing back towards the previous baseline (figure 7*c*). This initial acceleration of the conductance change may be due to the continued presence of BSO inside the cell, and the time required to wash it out, before the depleted intracellular glutathione levels can be replenished sufficiently to reduce intracellular redox and thus close the hemichannel.

Interestingly, removal of NAC revealed a slow and reversible conductance increase, suggesting that the native redox state of the HeLa cells is slightly oxidized compared with the highly reduced state induced by inclusion of NAC (figure 7*c,d*). The increase in conductance following removal of NAC also had a time constant some 20 times slower than the response of wild-type Cx26 to CO_2_ (figure 7*e*).

The residue 125 is near the intracellular loop and thus on the cytoplasmic side of the plasma membrane. The dynamics of hemichannel opening will therefore result from a combination of how quickly the agonist can permeate into the cell to reach the binding site on residue 125 and the propensity for the covalent reaction at residue 125 to occur. For the redox sensitivity, there is the additional complication that NAC and BSO act indirectly to change the intracellular concentration of glutathione, which then alters intracellular redox state to either make or break the disulfide bridge between C125 and C104 of adjacent subunits. It is therefore not surprising that the new agonist sensitivities that we have introduced into the mutated channels have considerably slower kinetics than the native sensitivity to CO_2_. Nevertheless, our results with BSO and NAC imply that glutathione metabolism is highly dynamic within the HeLa cells and is consistent with other observations of glutathione synthesis in these cells [12].

## 5. Discussion

To the best of our knowledge, this is the first time that novel agonist sensitivity has been introduced into connexin hemichannels. With our hypothesis-driven mutational strategy, we have converted wild-type Cx26 from a CO_2_-sensitive hemichannel to an NO/NO_2_^−^-sensitive hemichannel in Cx26^K125C^ and a redox-sensitive hemichannel in Cx26^K125C,R104C^ (figure 1). Crucially, wild-type Cx26 hemichannels do not exhibit sensitivity to either NO donors, NO_2_^−^ or intracellular redox. This contrasts with some other connexin and pannexin hemichannels that can be regulated by NO and intracellular redox [13–15]. Our results are important in two respects. First, they provide further support for our hypothesis that bridges involving residues 125 and 104 of adjacent subunits can gate the opening of Cx26. We initially proposed this hypothesis for the gating of Cx26 by CO_2_ [5]. By introducing mechanistically different bridges at the same location in the protein, we have shown that alternative agonists can open the hemichannel, providing they promote inter-subunit bridge formation between residues 125 and 104, even if this is via a different mechanism. Second, our new evidence further strengthens the concept of hemichannels as agonist-gated channels somewhat analogous to ligand-gated receptors. In a traditional ligand-gated receptor, the ligand binds and elicits a conformational change, which allows ion flow through the receptor channel. For hemichannels, the ligand or agonist binds to cause conformational change, and the opening of the hemichannel allows the fluxes of small molecules in addition to ions. ATP, an important physiological signalling molecule in many organ systems including the brain, is known to permeate connexin hemichannels [4,16,17]. Cx26 and related β connexins, which are opened by CO_2_ [4,5,8], and other hemichannels such as Cx37, Cx40 and Cx43, which are opened by NO [18], therefore provide mechanisms for physiologically important ligand-gated ATP release.

In wild-type Cx26, the available crystal structure [19] suggests that K125 and R104 of adjacent subunits are only about 6 Å apart. Once carbamylation has occurred this distance will fall to about 3 Å, which is suitable for formation of a salt bridge. In Cx26^K125C^ C125 is 10.5 Å from R104 in the neighbouring subunit (figure 1), and with SNO formation this distance would fall to about 7–8 Å. Ordinarily, this would be too far for interaction between the SNO group and R104. Similarly, in Cx26^K125C,R104C^ the two introduced cysteine residues would be nearly 16 Å apart—a gap far too large for disulfide bridge formation. Nevertheless, our data strongly suggest the formation of an SNO bridge between C125 and R104, and a disulfide bridge between C125 and C104. This implies considerable flexibility in the protein so that these residues can come into sufficient proximity to allow interaction and bond formation. Our elastic network models [5] based on the crystal structure [19] suggest that Cx26 is highly flexible with harmonic motions of the major alpha helices that could bring these residues into close apposition and permit bond formation. These data therefore provide additional support for our previous elastic network models. The large distance of these interactions may also contribute to their slow formation, and be an additional reason why NO_2_^−^ and intracellular redox cause rather slow channel opening.

Finally, we have generated molecules with new functionality that can, respectively, give NO/NO_2_^−^ and intracellular redox-mediated ATP release. The closest precedent to this change of agonist specificity for a receptor is the creation of the DREADD receptors from the muscarinic receptor [20]. DREADD receptors can be opened by the synthetic agonist clozapine-*N*-oxide, and have been highly useful analytical tools to test the roles of neuronal populations [21,22]. Our modified Cx26 hemichannels may conceivably be useful in the analysis and perturbation of NO and redox signalling.

## Supplementary Material

Data for Figures 3-7

## Data accessibility

The datasets supporting this article have been uploaded as part of the electronic supplementary material.
